# Comparison of Global Navigation Satellite System Devices on Speed Tracking in Road (Tran)SPORT Applications

**DOI:** 10.3390/s141223490

**Published:** 2014-12-08

**Authors:** Matej Supej, Ivan Čuk

**Affiliations:** 1 Department of Biomechanics, Faculty of Sport, University of Ljubljana, Ljubljana 1000, Slovenia; E-Mail: Ivan.Cuk@fsp.uni-lj.si; 2 Faculty of Mathematics, Natural Sciences and Information Technologies, University of Primorska, Koper 6000, Slovenia

**Keywords:** Global Positioning System, measuring devices, sport, vehicle, velocity

## Abstract

Global Naavigation Satellite Systems (GNSS) are, in addition to being most widely used vehicle navigation method, becoming popular in sport-related tests. There is a lack of knowledge regarding tracking speed using GNSS, therefore the aims of this study were to examine under dynamic conditions: (1) how accurate technologically different GNSS measure speed and (2) how large is latency in speed measurements in real time applications. Five GNSSs were tested. They were fixed to a car's roof-rack: a smart phone, a wrist watch, a handheld device, a professional system for testing vehicles and a high-end Real Time Kinematics (RTK) GNSS. The speed data were recorded and analyzed during rapid acceleration and deceleration as well as at steady speed. The study produced four main findings. Higher frequency and high quality GNSS receivers track speed at least at comparable accuracy to a vehicle speedometer. All GNSS systems measured maximum speed and movement at a constant speed well. Acceleration and deceleration have different level of error at different speeds. Low cost GNSS receivers operating at 1 Hz sampling rate had high latency (up to 2.16 s) and are not appropriate for tracking speed in real time, especially during dynamic movements.

## 1. Introduction

Positioning and tracking of people and transport vehicles by the Global Navigation Satellite System (GNSS) underwent a major expansion recently, especially after the arrival of smart phones, which almost as a rule have an in-built GNSS receiver. If the GNSS with the commercial name Global Positioning System (GPS) from the USA reigned in the global positioning in the past decades, lately a number of devices that use the synchronous Russian GNSS system named Globalnaja Navigacionnaja Sputnikovaja System (GLONASS) have rapidly expanded. The reason for the increasing number of devices using both GNSS systems is understandable, since the GLONASS expanded from 6 to 24 satellites in the past decade [1,2]. The increased number of satellites makes accurate position measurements more likely and at the same time enables the receiver to exclude satellites with poor signal, which improves the accuracy and reliability of positioning [1,3]. Moreover, many devices lately are compatible with the European GNSS system, Galileo, which is expected to become operational in 2019 and will be particularly significant in order to decrease the obstruction problem in Europe [4].

Most people are familiar with GNSS devices from applications for road navigation, although the technology roots are in the radio-navigation systems, *i.e.*, Long Range Navigation (LORAN), which was developed for the navigation of ships and aircrafts. Precisely because of the great need to determine the position of vehicles, vessels and aircrafts and to obtain their navigation information, most studies were made in connection with positioning accuracy [5–10], problems of obstructions [4], time to fix [11,12], *etc.* Despite the fact that GNSS technology uses satellites and their signals for positing, there are considerable differences among the available systems in:

- The number of frequencies they use from the satellites,
- The satellites they can track (e.g., GPS and/or GLONASS),
- The sampling frequency,
- The number of channels for tracking satellites,
- The capacity to correct position calculations, such as Satellite-Based Augmentation Systems (SBAS) or Real Time Kinematics (RTK) differentiation.

Consequently, several studies examined the impact of these technical specifications on the positioning accuracy [1,5,9,13,14]. In addition to positioning and navigating, GNSS systems are in practice almost as a standard used for testing cars, motorcycles, bicycles, *etc.* Due to increasing accessibility and accuracy such tests have greatly expanded in the sports in connection with various means of transport [7]. The reason of course is that sports requirements are set higher than ever and the differences between competitors or means of transport are decreasing. In most of these applications, the traveling speed of GNSS systems is usually one of the most important data which is obtained in different ways using different GNSS systems. In contrast to the normal differentiation of positions, some systems use the Doppler Effect to measure speed [15,16].

Previous research on the accuracy of speed measurements by GNSS systems has been primarily focused on human movement [17,18]. As far as we are aware, only one study addressed the problem of changing speed [13], while all others focused only on the accuracy of constant speed [8,9,14,16], although in some studies movement included circular motion [9,14]. In addition, no study analyzed systems that use the Doppler Effect to measure speed; phones with an integrated GNSS receiver; devices that use GPS and GLONASS satellites simultaneously, and none of them examined time latency of speed measurements in a variety of GNSS systems. Therefore, the aims of this study were to examine in dynamic conditions: (1) how accurate technologically different GNSS systems measure speed and (2) how large is latency of speed measurements in real time applications.

## 2. Experimental Section

### 2.1. Instruments

**Figure 1.**
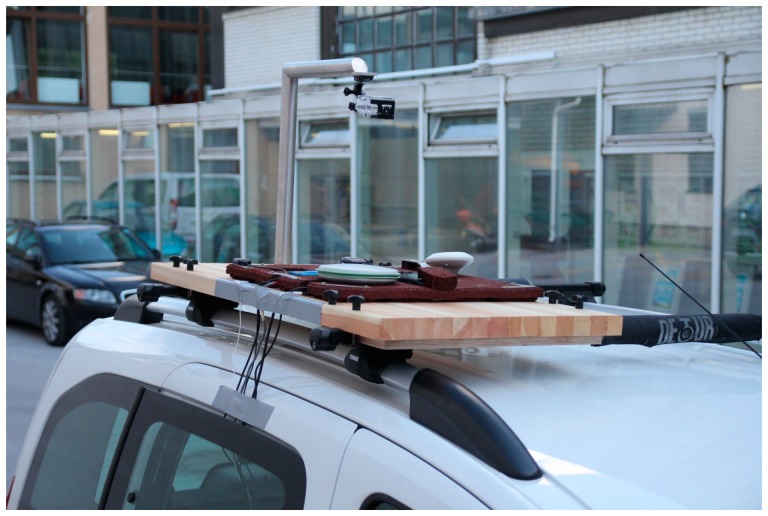
Custom-built platform for mounting GNSS devices and cameras on the roof rack of the car.

A Peugeot Partner Tepee passenger car was used for measuring purposes. A specially prepared platform that allowed mounting a variety of global navigation satellite systems and associated antennas was installed on the car's roof rack (Figure 1). The platform also had a special arm that allowed installation of a high definition wide-angle camera GoPro HD Hero 3 black (GoPro, Inc., San Mateo, CA, USA). The camera was positioned in such a way that it covered the displays of all GNSS devices simultaneously at a frequency of 30 Hz and recording resolution of 1080 p, which was sufficient for reading the displays.

Five different GNSS devices ranging from a simple consumer product to high-end high-frequency real time kinematics (RTK) GNSS devices have been used in the study:

1) Smart phone: HTC Sensation HD (HTC Co., Taoyuan City, Taiwan)
2) Wrist watch: Garmin Forerunner 305 (Garmin International Inc., Kansas City, KS, USA)
3) Handheld device: Locosys Genie GT-31 (LOCOSYS Technology Inc., Taipei, Taiwan)
4) Professional system for testing engines and vehicles: Racelogic VBOX 20 SX (Racelogic Ltd., Buckingham, UK) with an external antenna B3G02G (Inpaq Technology Co., Ltd, Taipei, Taiwan)
5) High-end geodetic Leica RTK GNSS system (Leica Geosystems, Heerbrugg, Switzerland) consisting of a rover and a reference station, which had equal hardware components: RTK GNSS receivers (Leica GX1230GG), Leica survey antennae (GLONASS/GPS AX1202 GG) and Leica Satelline 3AS radio modems. The reference station was set up at the appropriate Leica tripod.

In the text devices are abbreviated using the name of the manufacturer. The simplest device, the phone, was single-frequency, operating at a frequency of 1 Hz and was capable of tracking only GPS satellites; the most advanced Leica system was a dual frequency L1/L2 receiver, the system operated at 20 Hz in RTK mode and simultaneously tracked both GPS and GLONASS satellites. Selected technical specifications of the GNSS devices are shown in Table 1.

The HTC phone used the Torque Pro application [19] working in the Android v2.3 operating system for data recording. All other devices stored data with its standard built-in applications, either in internal memory (Garmin), or on the memory card (Locosys, Racelogic and Leica), and each at its maximum sampling rate.

**Figure 2.**
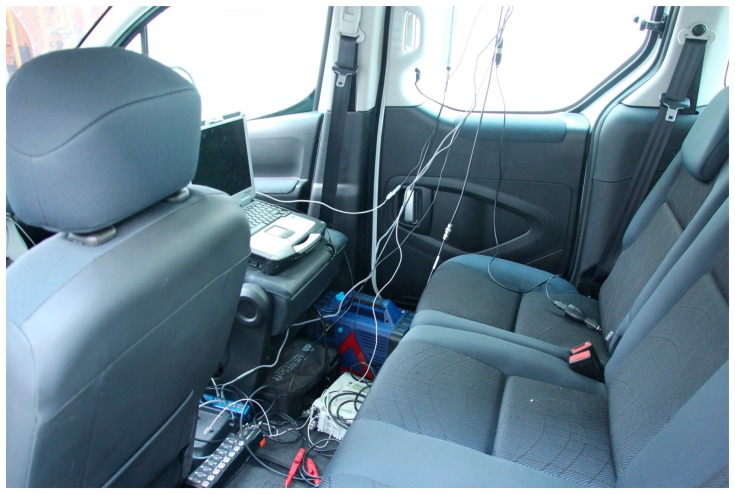
Laptop with the external analog-digital unit in the vehicle synchronously stored data from two GNSS systems and the vehicle speedometer.

In addition to previously described recording, the Leica and Racelogic were connected via the RS232 port to the combined digital-analog unit DEWE 43 (Dewesoft Ltd., Trbovlje, Slovenia). DEWE 43 was via standard CAN protocol and OBDII input associated with a vehicle computer (Figure 2). For synchronous recording of time, position and speed of the Leica and Racelogic; and speed from the vehicle speedometer, and to monitor these measurements in real time, the DEWE 43 was connected to a Panasonic Toughbook 31 laptop (Panasonic Corp., Osaka, Japan) equipped with Dewesoft X software (Dewesoft Ltd.). It should be noted that the Dewesoft system enabled synchronized recording of signals coming at different times and frequencies. As the HTC, Garmin and Locosys devices have no possibility of such a link, the latter were synchronized via visual information from the camera mounted on the roof rack, which filmed displays of all GNSS systems. Such camera view resembles the normal user view on the GNSS display which in this case showed the speed for each system. By comparing the reading on the display and the data values in the recorded data these systems were synchronized with an accuracy of 1/30 s. All GNSS devices used in the study provided speed (magnitude of velocity) as an output parameter; exact algorithms how the speeds are obtained in these devices are not revealed by the manufacturers.

### 2.2. Experiment Protocol

The experiment was carried out on a long straight road with no trees, houses or other buildings and the hills far away constituted an obstacle of below 5 degrees azimuth. Three sets of trials were performed:

- Rapid acceleration to speed of about 50 km/h and rapid deceleration
- Rapid acceleration to speed of about 70 km/h and rapid deceleration, and
- Driving at constant speed of 50 km/h using the cars inbuilt cruise control device.

**Figure 3.**
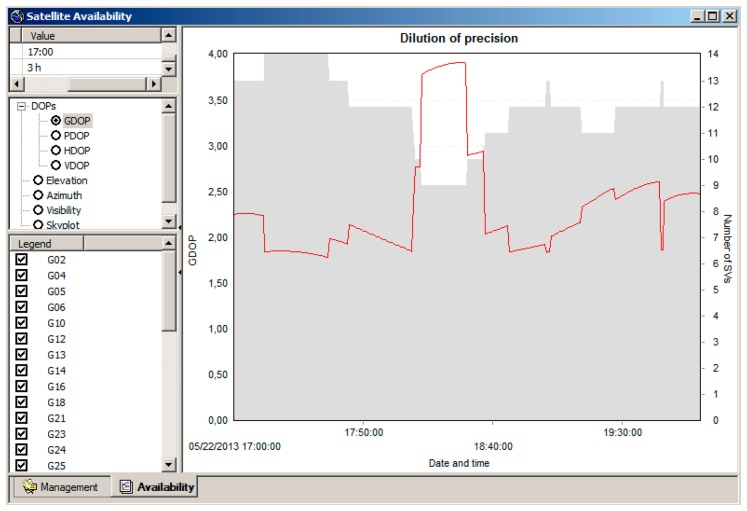
Number of visible satellites (SV) and geometric dilution of precision for the time of measurements.

Trials in set I and in set II were performed 12 and 14 times, respectively. Driving at constant speed (set III) was carried out once. The measurements took in total more than three hours. The measurement timing was planned with the Leica GeoOffice application, whereby it was verified that in this period of time, the constellations of satellites were such that some trials would have high satellite availability with a large number of visible satellites: 8× GPS and 7× GLONASS and low geometric dilution of precision (GDOP < 2), and other would have relatively low satellite visibility with a small number of visible satellites: 5× GPS and 4× GLONASS and a high GDOP ∼ 4 (Figure 3).

### 2.3. Data Processing

**Figure 4.**
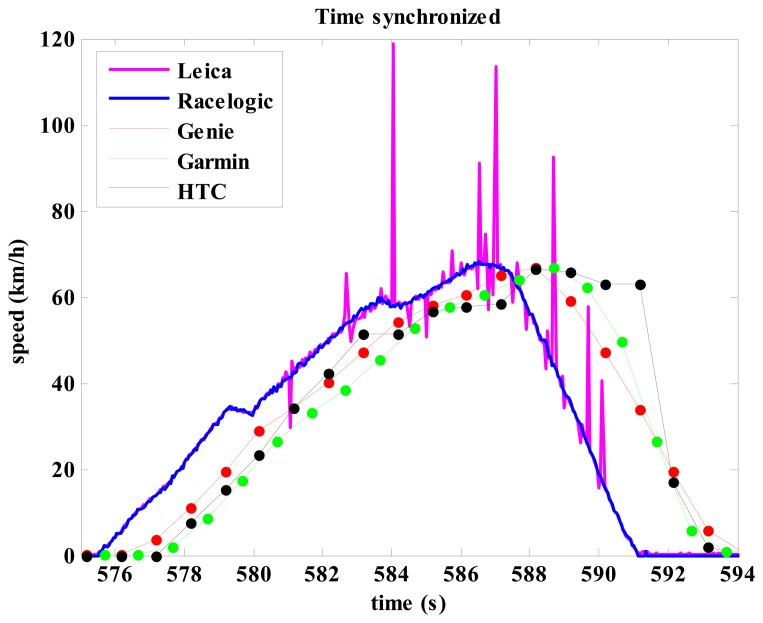
An example of time synchronized speed for all five GNSS devices with an obvious interference in the signal on the Leica system. Note: Leica and Racelogic had 20 Hz sampling rate (bold line) and other systems had 1 Hz sampling rate (full circles, thin line is drawn to help the reader).

First, all the data from all trials and from of all measuring devices were reviewed and two attempts in the first set of measurements and another attempt from the second set of measurements were eliminated, as there was obvious degradation of signal quality on the Leica system (Figure 4). The remaining trials were used for analysis. At first, the data from the Leica, Racelogic and vehicle speedometer, which were directly recorded to the laptop computer, were interpolated with a cubic spline function to 20 Hz data rate which enabled analysis of differences between vehicle speedometer and the two most advanced GNSS systems in the experiment (Leica and Racelogic). Among the remaining 23 trials one trial was randomly selected: it contained 2064 measured speeds. Depending on the vehicle speedometer, 784 measured speeds from in the selected trial had uniform speed, 899 measurements were recorded at the time of acceleration and 381 measurements at the time of deceleration. The criterion for uniform speed, acceleration and deceleration were speed records from the vehicle speedometer. For this part of the experiment another two variables were recorded, namely, the absolute difference in speed between the vehicle speedometer and the speed from: (1) Leica and (2) Racelogic. Thereafter a Bland Altman test [20,21] was used to analyze the compliance of the measurement process.

For the second step, the data was already synchronized for the Leica and Racelogic, the data for the other three GNSS systems (HTC, Garmin and Locosys) were synchronized by comparing the readings from the displays recorded via an external camera and the data recorded in the memory of devices. For this purpose, the video was played back step by step in order to find the frame where the speed value on the display of a GNSS device changed. The same value was thereafter found in the recorded data which were shifted in time according to the video time difference retrieved by reading the display from the professional GNSS system. The time carrier frequency was again 20 Hz and the data were interpolated in a similar way as the user can read the display on the device: the value remains constant until the next value appears (piecewise constant interpolating). For this part of the experiment, Leica speed measurements, *S_Leica_*, were used as a “golden standard”. Therefore, the speed data, *S_GNSS_*, from each of other four GNSS systems of the entire measurement was moved back and forth in time, Δ*t*, until a minimum in sum of the squares of the differences in speed between Leica and the system in Equation (1) was found numerically:

(1) $$\underset{\Delta t}{\min}\left(\sum_{i}{\left(S_{\text{Leica}}\left(t_{\text{Leica},i}\right)-S_{\text{GNSS}}\left(t_{\text{GNSS},i}+\Delta t\right)\right)}^{2}\right)$$

where the abbreviation *t_(Leica,GNSS),i_* stands for time for the examined golden standard system (Leica) and other four GNSS systems; and *i* is the increment in the sum. In this calculation the speed data recorded between individual trials (apparatus consisted of continuous data for the whole measurement time) were excluded. The difference in time between the devices synchronized in time and synchronized devices at speeds defined latency in speed of GNSS devices.

From these speed synchronized data two trials with acceleration up to speed 70 km/h were randomly chosen. A Bland Altman test was used to compare the Racelogic, Genie, Garmin and HTC against the Leica. In addition, t-tests between the various navigation systems while driving at a steady speed (the chosen trials had 47 and 100 speed measurements), acceleration (the chosen trials had 188 and 161 speed measurements) and deceleration (the chosen trials had 169 and 143 speed measurements) for each experiment separately were calculated. Finally Spearman's rank correlation between the two chosen trials were performed.

In the third step, the differences (pairwise t-test) and relationship (Spearman's rank correlation) at maximum speed were compared between all navigation systems at trials with accelerations up to speeds of 70 km/h (10 measurements) and 50 km/h (10 measurements).

In the fourth step, it was analyzed how the navigation systems were in line (pairwise t-test, Spearman's rank correlation) at constant speed of 50 km/h; the time interval of steady driving included 10 measurements.

In the end it was inspected how many satellites had been tracked during the measurements by the GNSS receivers used in this study. The mean value and standard deviation of tracked satellites was calculated.

The collected data were analyzed using Excel to calculate Bland Altman test (to define agreement between different GNSS systems). We used statistical program SPSS 22.0 to calculate Kolmogorov-Smirnov test (to define weather data is normally distributed), Pairwise t-tests (to define amount of differences between GNSS systems; in results section in Tables is used p_(t-test)_ just to mark significance of t-test without the data of average and standard deviation) and Spearman's rank-correlation coefficients (to define relation of results between GNSS systems). The level of significance was set to *p* < 0.05 and because there were considerably more significant results, the non-significant results were noted with asterix (*) throughout the manuscript.

## 3. Results

**Figure 5.**
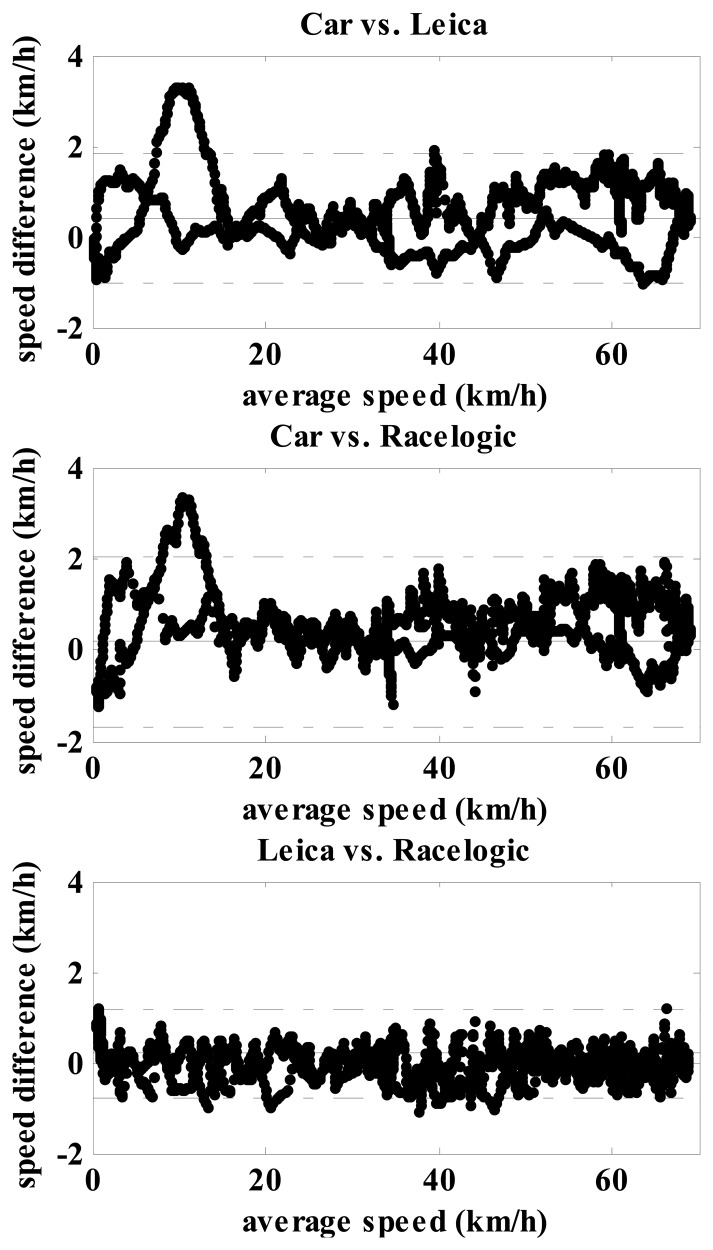
Bland Altman test of agreement between measured protocols. On x axis is average speed of both compared devices; on y axis is difference in speed between both devices; all data are presented in km/h. Full horizontal line represent the mean differences and the dash-dot line 1.96 SD (standard deviation) interval in each diagram.

First the speeds recorded by the Leica and the Racelogic were tested against the vehicle speedometer. Variables tested with Kolmogorov Smirnov were not normally distributed. Therefore pairwise sample t-tests and Spearman rank correlation were calculated. A comparison between the vehicle speedometer and the Leica and Racelogic showed there were some minor differences in speed measurements (Table 2). While speed was constant, the Leica and the vehicle speedometer did not differentiate significantly (0.31 km/h); it should be noted that vehicle speedometer measurements were obtained in 1 km/h precision (no decimals). The absolute difference between the vehicle speedometer and the Racelogic was slightly less than 1 km/h and statistically significant. The Leica and Racelogic had a similar difference from the vehicle speedometer during deceleration. The Racelogic and Leica demonstrated almost perfect correlation to the vehicle speedometer during acceleration and deceleration (Tables 2 and 3), therefore only systematic error of measurement was possible. The worst, but still high and significant was the correlation between the vehicle speedometer, the Racelogic and Leica during constant speed; however, they still share more than 50% of variance and the Racelogic was slightly better. A Bland Altman test (Figure 5) showed that Leica and Racelogic had high agreement between themselves. Agreement with the vehicle speedometer, the Leica and Racelogic showed slight problems at speeds at around 10 km/h where both systems differed from the vehicle speedometer by more than 3 km/h. The Leica and Racelogic were in agreement of less than 1.5 km/h within all measured speeds. According to the results of t-test, Spearman rank correlation, absolute differences with car and Bland Altman test it was decided to use the Leica as the golden standard for comparisons with other GNSS systems.

**Figure 6.**
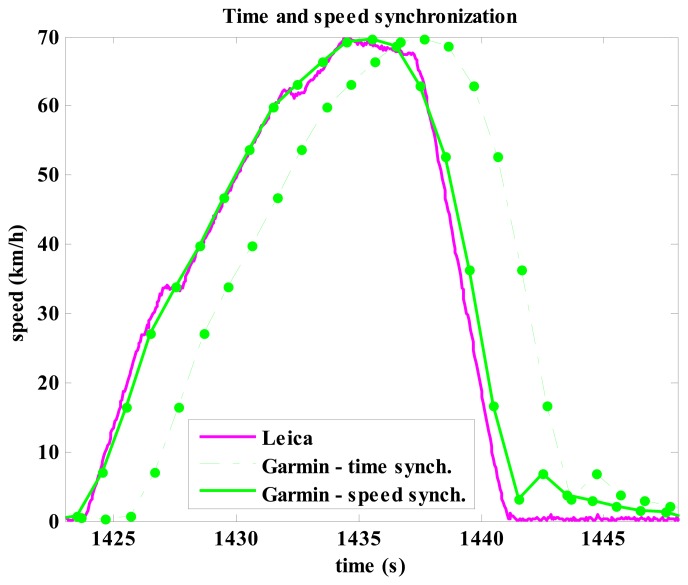
Garmin speed when time synchronized and speed synchronized against the reference system Leica for one selected trial. The speed latency was 2.16 s. Note: Leica had 20 Hz sampling rate (bold line) and Garmin had 1 Hz sampling rate (full circles, thin line is drawn to help the reader).

When comparing the Racelogic, Genie, Garmin and HTC devices against the Leica one, latency was the first parameter examined. Latency for speed measurement was 0.02 s, 1.45 s, 2.16 s, and 2.01 s for the Racelogic, Genie, Garmin and HTC, respectively. Speed latency for the Garmin is shown in Figure 6 for one selected measurement. The size of latency in practice results in wrong real time speed information, for example, the Garmin shows speed of 62.8 km/h when the vehicle is actually stationary. Similar discrepancy due to latency was observed in the results from the Genie and HTC, but the difference was slightly lower due to lower latency. The diagram in Figure 6 shows another speed error for the Garmin which occurred similarly on several other trials, but not consistently on all of them. When speed should drop down to zero, the speed retrieved from the Garmin actually jumped up to approximately 10 km/h and then slowly decreased in the next 6–7 s toward zero. A similar and less evident problem was also observed in the Genie, but there the speed dropped to zero in 1 s. The HTC didn't show such problems, but it had problems with calculating the speed at each time interval (each second). In many cases the speed at a given time was not calculated; instead, in the next one or two second an identical speed value appeared (Figure 4, see HTC speed for the time between 590 s and 592 s).

In Table 4 the most important result is pairwise t-test between pairs of GNSS systems during two different measurements showed in the one line before last signed with p_(t-test)_ where significance of t-test between pairs of two samples is calculated. Results of p_(t-test)_) show there were no significant differences between 10 pairs of GNSS systems within two measurements, meaning that GNSS systems worked consistently during constant speed, during acceleration and deacceleration. The bottom line shows Spearman rank correlation between both samples; Spearman rank correlation was significant and high during constant speed and acceleration. While during deacceleration Spearman rank correlation was low and not significant, mostly due to the Genie, as from one measurement to another pairwise t-test values changed from positive toward negative ones; there was also a change in the pair HTC and Garmin. The most similar values correspond to the Racelogic and HTC, where only during acceleration in the second measurement were differences observed.

Results of statistics for differences between GNSS systems at maximum speed of 70 km/h and 50 km/h are shown in Table 5. In the range of 50 km/h all GNSS systems measured significantly different maximum speed with small mean differences ranging from 0.46 km/h to 1.35 km/h (0.9%–2.7%). In the range of 70 km/h not all navigations system pairs were significantly different, and again with small mean differences ranging from 0.14 km/h to 1.85 km/h (0.2%–2.6%). All Spearman rank correlations were significant and high, but at speed of 50 km/h were higher.

When measured constant speed at 50 km/h, very high significant Spearman rank correlations were found (Table 6). In a majority there were non-significant paired samples t-tests; significant once were those between the pairs Racelogic–Garmin, Genie–HTC; Genie–Leica and HTC–Garmin (Table 6). It can be concluded that with a long time span of constant velocity all GNSS systems reach agreement.

**Figure 7.**
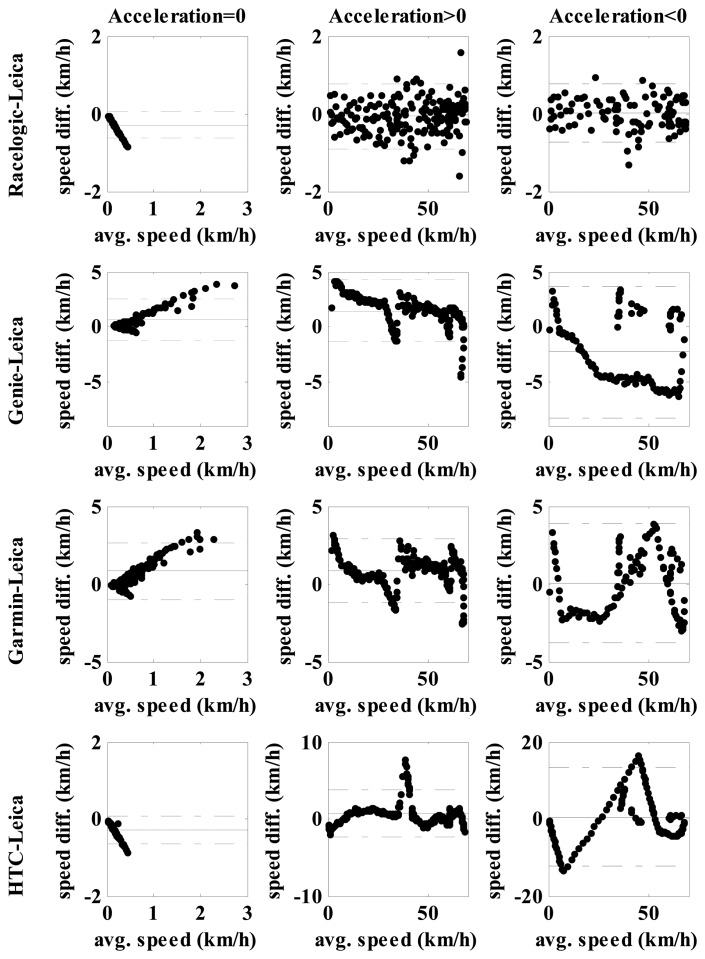
Bland Altman test of agreement between the Leica and other GNSS devices. On x axis is average speed of both compared devices; on y axis is difference in speed between both devices. The first row shows data for steady speed, the second for acceleration and the third deceleration. Full horizontal line represent the mean differences and the dash-dot line 1.96 SD (standard deviation) interval in each diagram. Note that the vertical scale change sometimes in order to improve the visibility of the diagrams.

Bland Altman test (Figure 7) showed very high agreement in measured values in all conditions when testing the Racelogic *versus* the Leica. During constant speed there were small linear dependences and differences of up to 1 km/h in speeds up to 0.5 km/h. While accelerating or decelerating their error is random and in general smaller than 1 km/h. We can conclude that both systems were measuring same speed, as previously stated, in agreement with the velocity speedometer.

Bland Altman test (Figure 7) showed that the Genie, Garmin and HTC had different relationships with the Leica in contrast to when the Racelogic was compared to the Leica.

*Pair Genie-Leica:*

- At constant speed up to 3 km/h the Genie had “linearly” higher values up to 4 km/h;
- While accelerating at higher speed the difference was decreasing (from 4 km/h toward 0 km/h) and in some cases even negative where the Leica retrieved higher values (the Genie underestimated speed), range was ±5 km/h;
- A similar trend was observed during deceleration where the range of differences between both systems was up to 10 km/h.

*Pair HTC-Leica:*

- At constant speed the differences were small (<1 km/h) at speeds of less than 0.5 km/h;
- While accelerating the average difference was ±2 km/h and in the range of 40 km/h it rose up to 8 km/h with the HTC having higher values;
- While decelerating a zig-zag curve was observed, with very high differences up to ±15 km/h.

*Pair Garmin-Leica:*

- At constant speed up to 3 km/h the Garmin had “linearly” higher values up to 4 km/h;
- While accelerating a line-like pattern was observed similar to the pair Genie-Leica; only the range of differences was lower ±3 km/h;
- During deceleration the range was lower compared to the HTC and Genie (±4 km/h) and again it varied during different speeds.

**Figure 8.**
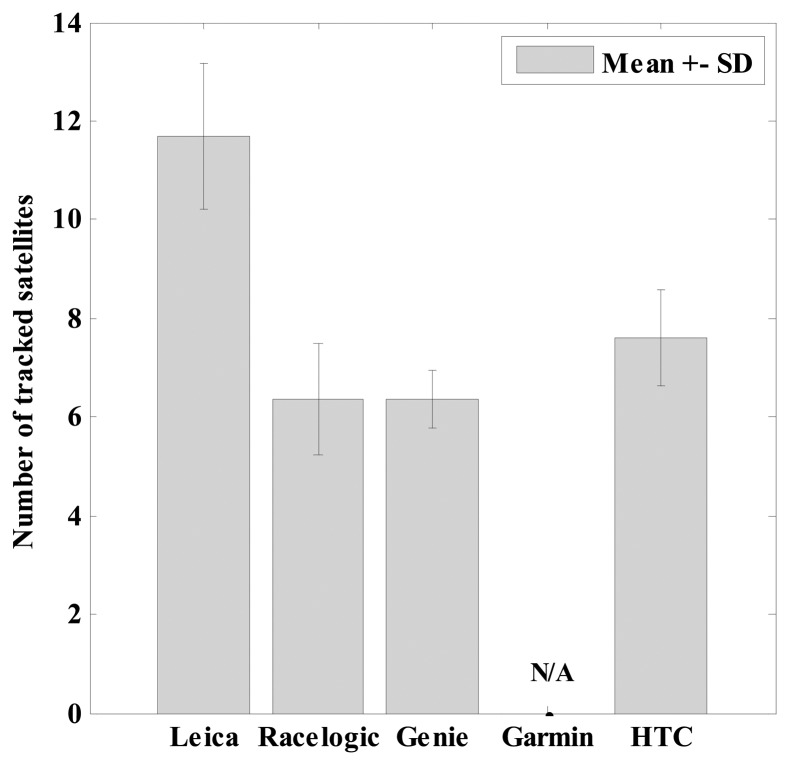
Mean number of tracked satellites (bars) for each GNSS device during the measurements and the corresponding standard deviations (SD) (error bars). Note that the number of tracked satellites for Garmin was not available (N/A).

During measurements, the GNSS systems tracked a different number of satellites. As can be observed from Figure 8, the largest number of satellites were tracked by the Leica which simultaneously uses GPS and GLONASS satellites. The Garmin does not provide any insight into the number of tracked satellites and the rest of the GNSS systems using only GPS satellites demonstrated similar numbers of tracked satellites during the measurements.

## 4. Discussion

The main findings of the study were: (1) higher frequency and high quality GNSS receivers such as the Leica (20 Hz RTK GNSS) and Racelogic (20 Hz) track speed with accuracy at least comparable to the vehicle speedometer; (2) all GNSS systems included in the test well measured maximum speed and movement at constant speed; (3) acceleration and deceleration were well in agreement only in the Leica and Racelogic; and (4) low cost GNSS receivers operating at 1 Hz sampling rate had a high speed latency and are not appropriate for tracking speed in real time during dynamic movements.

A vehicle speedometer and the two highest quality GNSS receivers, Leica and Racelogic, were compared first. It should be noted, that according to the regulations established by the United Nations Economic Commission for Europe (Transport Division–Vehicle Regulations) the passenger car speedometer must indicate speed which is never less than actual speed. In practice, modern cars display at least 2 or 3 km/h higher speed than actually measured with the inbuilt vehicle speedometer. This is important as the Dewesoft X system retrieves the “correct” speed value from vehicle computer and not the regulated one. The comparison results showed that the Leica and Racelogic GNSS measured speed statistically equivalently as the vehicle speedometer, which was for the purpose of this study especially important for examining accelerations and decelerations. The disadvantage of the Racelogic was that it had a trigger which at low speeds threw values to 0 km/h and somewhat spoiled velocities at lower speeds. However, this trigger could be useful for measurements/tests, but it can create problems where it is important to determine whether the vehicle or the athlete is stationary. Leica, on the other hand, had some insignificant noise when stationary, but had more trouble with good reception of signals from the satellites, although it has the most advanced receiver, method of operation and tracked in average the highest number of satellites (Figure 8). Obviously, the operation of RTK, which uses measurements of the phase of the signal′s carrier wave, needed higher quality signal compared to the normal mode of GNSS measurement, where the receiver's task is easier and needs to match the pseudorandom binary sequence of the signal to the “same” internally generated version of the information content. In the current study the loss of trials was quite high for the Leica: on average, 11.5% of trials had measurement data with some noise in speed which is surprisingly greater compared to other studies, for example, in a difficult alpine skiing environment [10,22]. Nevertheless, from the user perspective, it is important to guarantee the continuity of satellite visibility in dynamic applications when using systems operating in RTK mode in order to minimize the possibilities where a loss of lock on the internal tracking loops can frequently occur.

The Leica and Racelogic GNSS receivers had a higher refresh rate (20 Hz) compared to the speedometer (6 Hz) and in contrast to the speedometer displayed/recorded decimal places. Among these two GNSS devices the Leica expectedly outperformed the Racelogic after three “noisy” trails for Leica were excluded. Despite the fact that in our selected trials the vehicle speedometer demonstrated practically equivalent speed as the Leica, the problems of speedometer accuracy are well known in practice. An interesting discrepancy in speed was detected at low velocities in this comparison which can be attributed to the speedometer, as the two high quality GNSS systems working completely differently showed identical speed profile in this region. All this supports the decision that Leica served as a “golden standard” for further device comparisons.

From the comparison tests of GNSS devices against the Leica it was found that receivers running at 1 Hz sampling rate, such as GPS receiver built in a smart phone (HTC) and in handheld receivers (Garmin and Genie), performed well in constant speed and relatively well in maximum speed (significant but small difference compared to the golden standard). On the other hand, these GNSS systems had quite large latency in speed measurements. Speed data were delayed in the worst case by more than 2 s which can produce completely wrong speed information in real time at high accelerations/decelerations. As shown in Figure 6, the vehicle or the athlete could be already standing still and the GNSS device would still show maximum speed. At the same time they also have a relatively low frequency of 1 Hz refresh rate which is not favorable for measurements, for example in moto-sports or alpine skiing where the pace of speed changes is very high (note that in alpine skiing slalom a typical turn takes less than a second, therefore such a device would measure only one speed data per turn) [22].

Low cost GNSS systems in the current study demonstrated also some other problems in measuring speed as observed in Figures 4 and 6: speed does not drop to zero when stopped after rapid deceleration, the receivers didn't calculate speed in all time stamps (HTC). The statistical tests (Table 4 and Figure 7) also showed quite large systematic errors for low cost systems in movements where accelerations and decelerations were present which can't be the consequence of latency since the data for this test had been speed synchronized beforehand. The HTC, Garmin, Genie are intended for personal use, for some it is even highlighted that they are designed for sports activities, and one would expect that the lower speed range from 0–10 km/h (jogging) and in the range up to 30 km/h (recreational cycling) should be accurate enough to plan training. And, unfortunately, they were shown to be also in these areas with very large speed errors. Still, the Genie which uses the Doppler effect demonstrated the best performance among the 1 Hz GNSS systems, Garmin was slightly worse in acceleration/deceleration and the smart phone (HTC) had the greatest problems in tracking speed.

Therefore, despite the great potential of GNSS systems in sport practice and research, other technologies such as the Doppler radar, laser guns, photogrammetry based kinematic systems, accelerometers are still used to evaluate athletes speed [23–25]. However, none of the mentioned technologies can cover a large measurement area outdoor and therefore there is a need for technologies that can provide reliable and valid speed measurement and position information over a large capture volume which can be obtained by high-end GNSS systems [10,26] and, as shown in the current study, also by a high frequency SBAS systems such as the Racelogic, which is in comparison with RTK GNSS less demanding to perform measurements, more affordable and lighter.

The main limitation of the study was that it is virtually impossible to include all type of GNSS systems, especially due to a very rapid development of new technologies and their advances. However, the study incorporated a large variety of devices ranging from smart phones to high-end RTK GNSS devices and showed that low cost and low frequency systems suffered from similar problems and that the differences in terms of tracking speed are relatively small in high end systems. Another limitation of the study was the use of a vehicle speedometer for the first evaluation; however, it was basically used only to support the decision that the Leica can be used as a golden standard with insignificant latency (not found in this study, in comparison with the vehicle speedometer) and insignificant difference in tracking speed in dynamic conditions. A minor limitation of the study was also the time and place of measurements which could not take into account all possible conditions, despite the fact that it was well planned ahead to incorporate favorable and unfavorable satellite visibility.

## 5. Conclusions

Low-cost receivers with 1 Hz are only conditionally useful for tracking speeds in elite sports; and this is especially valid where speed is relatively steady. Even fewer are useful in (trans)sport movements for speed monitoring in real time, as they in addition have large latency.

Professional GNSS receivers such as the Leica and Racelogic track speed equally or even more accurately compared to the vehicle speedometer. Having a higher sampling rate compared to the vehicle speedometer (vehicle speedometer in the current study) they can be used to track speed of highly dynamic transport. At the highest level of speed accuracy and especially when combined with accurate positioning it is beneficial to use high-end RTK GNSS systems, emphasizing that the probability for loss of measurements is higher. On the other hand, a high-frequency system using only standard SBAS corrections (Racelogic) served only a minimally lower rate of measurements showing a great potential for speed measurements.

According to this study results it is important to point out that each new GNSS system should be tested for its intended use, since different systems suffer different problems to different extents. New GNSS systems should in particular be well examined for latency if they are meant to be used in real time applications, and for acceleration/deceleration if they are intended to be used is in dynamic movements.

## Figures and Tables

**Table 1.** Selected technical specifications of the GNSS devices. Legend: GNSS—Global Navigation Satellite System; GPS—Global Positioning System; GLONASS—Globalnaja Navigacionnaja Sputnikovaja System; A-GPS—Assisted GPS; SBAS—Satellite-Based Augmentation System; RTK—Real Time Kinematics.

**Device**	**GNSS Satellites**	**Doppler Effect**	**Frequency**	**Processing Mode**	**Sampling Rate (Hz)**	**Receiver's Number of Channels**	**Latency (ms)**	**Antenna Type**
HTC	GPS	No	L1	A-GPS	1	?	?	Internal
Garmin	GPS	No	L1	SBAS	1	12	?	Internal
Locosys	GPS	Yes	L1	SBAS	1	20	?	Internal
Racelogic	GPS	No	L1	SBAS	20	20	41.5	External
Leica	GPS + GLONASS	No	L1/L2	RTK	20	72	20	External

**Table 2.** Descriptive statistics for vehicle speedometer (Car), Racelogic, Leica, absolute difference Car-Racelogic, absolute difference Car-Leica (km/h). Legend: N—sample size, XA—mean, SE—standard error.

**Drive Type**	**N**	**Car XA ± SE**	**Racelogic XA ± SE**	**Leica XA ± SE**	**ABS (Car-Racelogic) XA ± SE**	**ABS (Car-Leica) XA ± SE**
All conditions	2064	30.1 ± 0.56	29.9 ± 55	29.7 ± .55	0.83 ± 0.01	0.60 ± 0.01
Acceleration = 0	784	15.4 ± 0.92	16.0 ± .89	15.4 ± .90	0.92 ± 0.00	0.31 ± 0.01
Acceleration > 0	899	40.5 ± 0.66	39.6 ± .66	39.6 ± .66	0.90 ± 0.02	0.93 ± 0.02
Acceleration < 0	391	36.0 ± 1.10	35.7 ± 1.12	35.9 ± 1.12	0.48 ± 0.02	0.43 ± 0.02

**Table 3.** Significance of pairwise sample t-test (lower triangle) and Spearman rank correlations (upper triangle) between vehicle speedometer (Car), Racelogic and Leica.

**All Condition**					

**Spearman Rank p_(t-test)_**	**Car**	**RACELOGIC**	**LEICA**	**ABS (Car-RACELOGIC)**	**ABS (Car-LEICA)**
Car		0.99	0.99	−0.22	0.52
RACELOGIC	0.00		0.98	−0.20	0.51
LEICA	0.00	0.00		−0.23	0.55
ABS(Car-RACELOGIC)					0.33
ABS(Car-GNSS1)				0.00	

**Acceleration = 0**					

**Spearman Rank p_(t-test)_**	**Car**	**RACELOGIC**	**LEICA**	**ABS (Car-RACELOGIC)**	**ABS (Car-LEICA)**

Car		0.84	0.79	−0.42	0.64
RACELOGIC	0.00		0.63	−0.02 *	0.50
LEICA	0.92 *	0.00		−0.39	0.64
ABS (Car-RACELOGIC)					−0.23
ABS(Car-GNSS1)				0.00	

**Acceleration > 0**					

**Spearman Rank p_(t-test)_**	**Car**	**RACELOGIC**	**LEICA**	**ABS (Car-RACELOGIC)**	**ABS (Car-LEICA)**

Car		1.00	1.00	0.18	0.23
RACELOGIC	0.00		1.00	0.17	0.23
LEICA	0.00	0.00		0.18	0.23
ABS (Car-RACELOGIC)					0.82
ABS(Car-GNSS1)				0.00	

**Acceleration < 0**					

**Spearman Rank p_(t-test)_**	**Car**	**RACELOGIC**	**LEICA**	**ABS (Car-RACELOGIC)**	**ABS (Car-LEICA)**

Car		1.00	1.00	−0.33	−0.08 *
RACELOGIC	0.00		1.00	−0.33	−0.09 *
LEICA	0.00	0.00		−0.33	−0.09 *
ABS (Car-RACELOGIC)					0.27
ABS(Car-GNSS1)				0.00	

* non-significant correlation or t-test

**Table 4.** Significance of pairwise sample t-test (p_(t-test)_) and Spearman rank correlation (bottom two lines) among GNSS systems for two selected measurements; acceleration = 0; acceleration > 0; acceleration < 0 (columns show values of pairwise t-test between pairs of GNSS systems for each sample).

**Pair**	**Acceleration = 0**		**Acceleration > 0**		**Acceleration < 0**	
		
	**Sample 1**	**Sample 2**	**Sample 1**	**Sample 2**	**Sample 1**	**Sample 2**
Racelogic–Genie	−7.29	−9.73	−8.73	−11.01	−5.82	4.72
Racelogic–HTC	0.46 *	−1.00 *	0.51 *	−4.77	−0.68 *	−1.19 *
Racelogic–Garmin	−13.58	−12.34	−4.79	−9.23	−8.55	−2.30
Racelogic–Leica	−8.86	−17.67	1.45 *	0.79 *	−1.63 *	−3.29
Genie–HTC	7.56	9.73	4.77	4.99	3.81	−3.36
Genie–Garmin	3.10	−2.72	7.83	8.04	4.10	−7.05
Genie–Leica	6.83	6.84	9.40	11.78	5.58	−5.53
HTC–Garmin	−14.76	−12.34	−1.89	−1.22 *	−2.64	0.45 *
HTC–Leica	−7.55	−17.43	−0.38 *	5.04	0.47 *	0.94 *
Garmin–Leica	11.50	8.98	5.20	9.82	7.57	1.63 *
p_(t-test)_	0.10 *		0.94 *		0.46 *	
Spearman Rank Correlation	0.91		0.91		−0.36 *	

* not significant at *p* < 0.05.

**Table 5.** Significance of pairwise sample t-test (p_(t-test)_) and Spearman rank correlations between GNSS systems at maximum speed of 70 km/h and 50 km/h.

**70km/h**		**Genie**	**HTC**	**Garmin**	**Leica**
p_(t-test)_	Racelogic	0.00	0.00	0.00	0.69 *
	Genie		0.03	0.01	0.00
	HTC			0.39 *	0.00
	Garmin				0.00
Spearman	Racelogic	0.95	0.80	0.90	0.98
Rank	Genie		0.81	0.86	0.97
Correlation	HTC			0.59	0.79
	Garmin				0.90

**50 km/h**		**Genie**	**HTC**	**Garmin**	**Leica**

p_(t-test)_	Racelogic	0.00	0.00	0.00	0.02
	Genie		0.01	0.00	0.00
	HTC			0.00	0.00
	Garmin				0.09 *
Spearman	Racelogic	0.95	0.94	0.96	0.95
Rank	Genie	0.97	0.99	0.99
Correlation	HTC			0.96	0.97
	Garmin				0.99

* t-test is not significant at *p* < 0.05.

**Table 6.** Significance of pairwise sample t-test (p_(t-test)_) and Spearman rank correlations between GNSS systems maintaining speed of 50 km/h

		**Genie**	**HTC**	**Garmin**	**Leica**
p_(t-test)_	Racelogic	0.07 *	0.25 *	0.00	0.06 *
	Genie		0.01	0.13 *	0.00	
	HTC			0.04	0.08 *
	Garmin				0.07 *
Spearman	Racelogic	0.95	0.99	0.99	0.99
Rank	Genie		0.97	0.99	0.99	
Correlation	HTC			0.99	0.99
	Garmin				0.99	

* t-test is not significant at *p* < 0.05.
